# Supplementary material for: Iterative improvement in the automatic modular design of robot swarms
Source: PeerJ Comput Sci. 2020 Dec 7;6:e322. doi: 10.7717/peerj-cs.322 (PMC7924708; doi:10.7717/peerj-cs.322)
Supplement: Supplemental Information 3 [file peerj-cs-06-322-s003.zip › argos3/doc/api/standalone/a00550_source.html]

ARGoS: plugins/robots/mini-quadrotor/simulator/miniquadrotor\_entity.cpp Source File


- Main Page
- Related Pages
- Namespaces
- Classes
- Files

- File List
- File Members

# plugins/robots/mini-quadrotor/simulator/miniquadrotor\_entity.cpp

Go to the documentation of this file.

```
00001 
00007 #include "miniquadrotor_entity.h"
00008 
00009 #include <argos3/core/simulator/space/space.h>
00010 #include <argos3/core/simulator/entity/controllable_entity.h>
00011 #include <argos3/core/simulator/entity/embodied_entity.h>
00012 #include <argos3/plugins/simulator/entities/rotor_equipped_entity.h>
00013 
00014 namespace argos {
00015 
00016    /****************************************/
00017    /****************************************/
00018 
00019    static const Real ARM_LENGTH = 0.63f;
00020 
00021    /****************************************/
00022    /****************************************/
00023 
00024    CMiniQuadrotorEntity::CMiniQuadrotorEntity() :
00025       CComposableEntity(NULL),
00026       m_pcControllableEntity(NULL),
00027       m_pcEmbodiedEntity(NULL),
00028       m_pcRotorEquippedEntity(NULL) {
00029    }
00030 
00031    /****************************************/
00032    /****************************************/
00033 
00034    CMiniQuadrotorEntity::CMiniQuadrotorEntity(const std::string& str_id,
00035                                               const std::string& str_controller_id,
00036                                               const CVector3& c_position,
00037                                               const CQuaternion& c_orientation) :
00038       CComposableEntity(NULL, str_id),
00039       m_pcControllableEntity(NULL),
00040       m_pcEmbodiedEntity(NULL),
00041       m_pcRotorEquippedEntity(NULL) {
00042       try {
00043          /*
00044           * Create and init components
00045           */
00046          /*
00047           * Embodied entity
00048           * Better to put this first, because many other entities need this one
00049           */
00050          m_pcEmbodiedEntity = new CEmbodiedEntity(this, "body_0", c_position, c_orientation);
00051          AddComponent(*m_pcEmbodiedEntity);
00052          /* Rotor equipped entity */
00053          m_pcRotorEquippedEntity = new CRotorEquippedEntity(this, "rotors_0", 4);
00054          m_pcRotorEquippedEntity->SetRotor(0, CVector3(ARM_LENGTH, 0.0f, 0.0f));
00055          m_pcRotorEquippedEntity->SetRotor(1, CVector3(0.0f, ARM_LENGTH, 0.0f));
00056          m_pcRotorEquippedEntity->SetRotor(2, CVector3(-ARM_LENGTH, 0.0f, 0.0f));
00057          m_pcRotorEquippedEntity->SetRotor(3, CVector3(0.0f, -ARM_LENGTH, 0.0f));
00058          AddComponent(*m_pcRotorEquippedEntity);
00059          /* Controllable entity
00060             It must be the last one, for actuators/sensors to link to composing entities correctly */
00061          m_pcControllableEntity = new CControllableEntity(this, "controller_0");
00062          AddComponent(*m_pcControllableEntity);
00063          m_pcControllableEntity->SetController(str_controller_id);
00064          /* Update components */
00065          UpdateComponents();
00066       }
00067       catch(CARGoSException& ex) {
00068          THROW_ARGOSEXCEPTION_NESTED("Failed to initialize entity \"" << GetId() << "\".", ex);
00069       }
00070    }
00071 
00072    /****************************************/
00073    /****************************************/
00074 
00075    void CMiniQuadrotorEntity::Init(TConfigurationNode& t_tree) {
00076       try {
00077          /*
00078           * Init parent
00079           */
00080          CComposableEntity::Init(t_tree);
00081          /*
00082           * Create and init components
00083           */
00084          /*
00085           * Embodied entity
00086           * Better to put this first, because many other entities need this one
00087           */
00088          m_pcEmbodiedEntity = new CEmbodiedEntity(this);
00089          AddComponent(*m_pcEmbodiedEntity);
00090          m_pcEmbodiedEntity->Init(GetNode(t_tree, "body"));
00091          /* Rotor equipped entity */
00092          m_pcRotorEquippedEntity = new CRotorEquippedEntity(this, "rotors_0", 4);
00093          m_pcRotorEquippedEntity->SetRotor(0, CVector3(ARM_LENGTH, 0.0f, 0.0f));
00094          m_pcRotorEquippedEntity->SetRotor(1, CVector3(0.0f, ARM_LENGTH, 0.0f));
00095          m_pcRotorEquippedEntity->SetRotor(2, CVector3(-ARM_LENGTH, 0.0f, 0.0f));
00096          m_pcRotorEquippedEntity->SetRotor(3, CVector3(0.0f, -ARM_LENGTH, 0.0f));
00097          AddComponent(*m_pcRotorEquippedEntity);
00098          /* Controllable entity
00099             It must be the last one, for actuators/sensors to link to composing entities correctly */
00100          m_pcControllableEntity = new CControllableEntity(this);
00101          AddComponent(*m_pcControllableEntity);
00102          m_pcControllableEntity->Init(GetNode(t_tree, "controller"));
00103          /* Update components */
00104          UpdateComponents();
00105       }
00106       catch(CARGoSException& ex) {
00107          THROW_ARGOSEXCEPTION_NESTED("Failed to initialize entity \"" << GetId() << "\".", ex);
00108       }
00109    }
00110 
00111    /****************************************/
00112    /****************************************/
00113 
00114    REGISTER_ENTITY(CMiniQuadrotorEntity,
00115                    "mini-quadrotor",
00116                    "Carlo Pinciroli [ilpincy@gmail.com]",
00117                    "1.0",
00118                    "The mini-quadrotor robot, developed at UPenn.",
00119                    "The mini-quadrotor is a fluing robot developed at UPenn by Vijay Kumar's group.\n"
00120                    "It is a very agile robot, able to perform fast and precise movements.\n\n"
00121                    "REQUIRED XML CONFIGURATION\n\n"
00122                    "  <arena ...>\n"
00123                    "    ...\n"
00124                    "    <mini-quadrotor id=\"mq0\">\n"
00125                    "      <body position=\"0.4,2.3,2.0\" orientation=\"45,0,0\" />\n"
00126                    "      <controller config=\"mycntrl\" />\n"
00127                    "    </mini-quadrotor>\n"
00128                    "    ...\n"
00129                    "  </arena>\n\n"
00130                    "The 'id' attribute is necessary and must be unique among the entities. If two\n"
00131                    "entities share the same id, initialization aborts.\n"
00132                    "The 'body/position' attribute specifies the position of the bottom point of the\n"
00133                    "mini-quadrotor in the arena. When the robot is untranslated and unrotated, the\n"
00134                    "bottom point is in the origin and it is defined as the middle point between\n"
00135                    "the two wheels on the XY plane and the lowest point of the robot on the Z\n"
00136                    "axis, that is the point where the wheels touch the floor. The attribute values\n"
00137                    "are in the X,Y,Z order.\n"
00138                    "The 'body/orientation' attribute specifies the orientation of the\n"
00139                    "mini-quadrotor. All rotations are performed with respect to the bottom point.\n"
00140                    "The order of the angles is Z,Y,X, which means that the first number corresponds\n"
00141                    "to the rotation around the Z axis, the second around Y and the last around X.\n"
00142                    "This reflects the internal convention used in ARGoS, in which rotations are\n"
00143                    "performed in that order. Angles are expressed in degrees. When the robot is\n"
00144                    "unrotated, it is oriented along the X axis.\n"
00145                    "The 'controller/config' attribute is used to assign a controller to the\n"
00146                    "mini-quadrotor. The value of the attribute must be set to the id of a previously\n"
00147                    "defined controller. Controllers are defined in the <controllers> XML subtree.\n\n"
00148                    "OPTIONAL XML CONFIGURATION\n\n"
00149                    "None.\n\n"
00150                    ,
00151                    "Under development"
00152       );
00153 
00154    /****************************************/
00155    /****************************************/
00156 
00157    REGISTER_STANDARD_SPACE_OPERATIONS_ON_COMPOSABLE(CMiniQuadrotorEntity);
00158 
00159    /****************************************/
00160    /****************************************/
00161 
00162 }
```

---

Generated on 10 Jul 2018 for ARGoS by 
 1.6.1
